# Supplementary figures and images for: Rare SH2B3 coding variants in lupus patients impair B cell tolerance and predispose to autoimmunity
Source: J Exp Med. Author manuscript; Available in PMC 2024 May 30. (PMC10901239; doi:10.1084/jem.20221080)

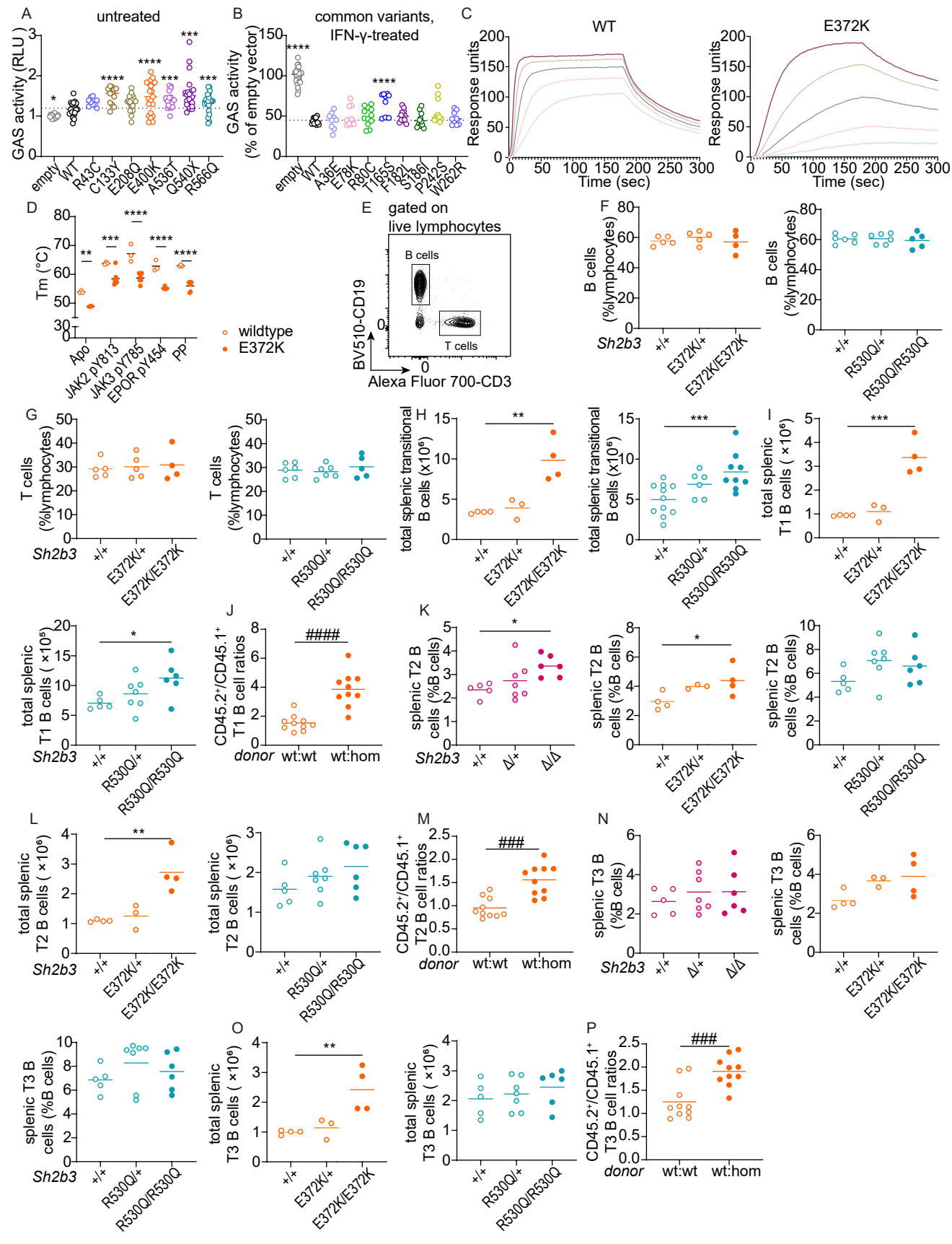

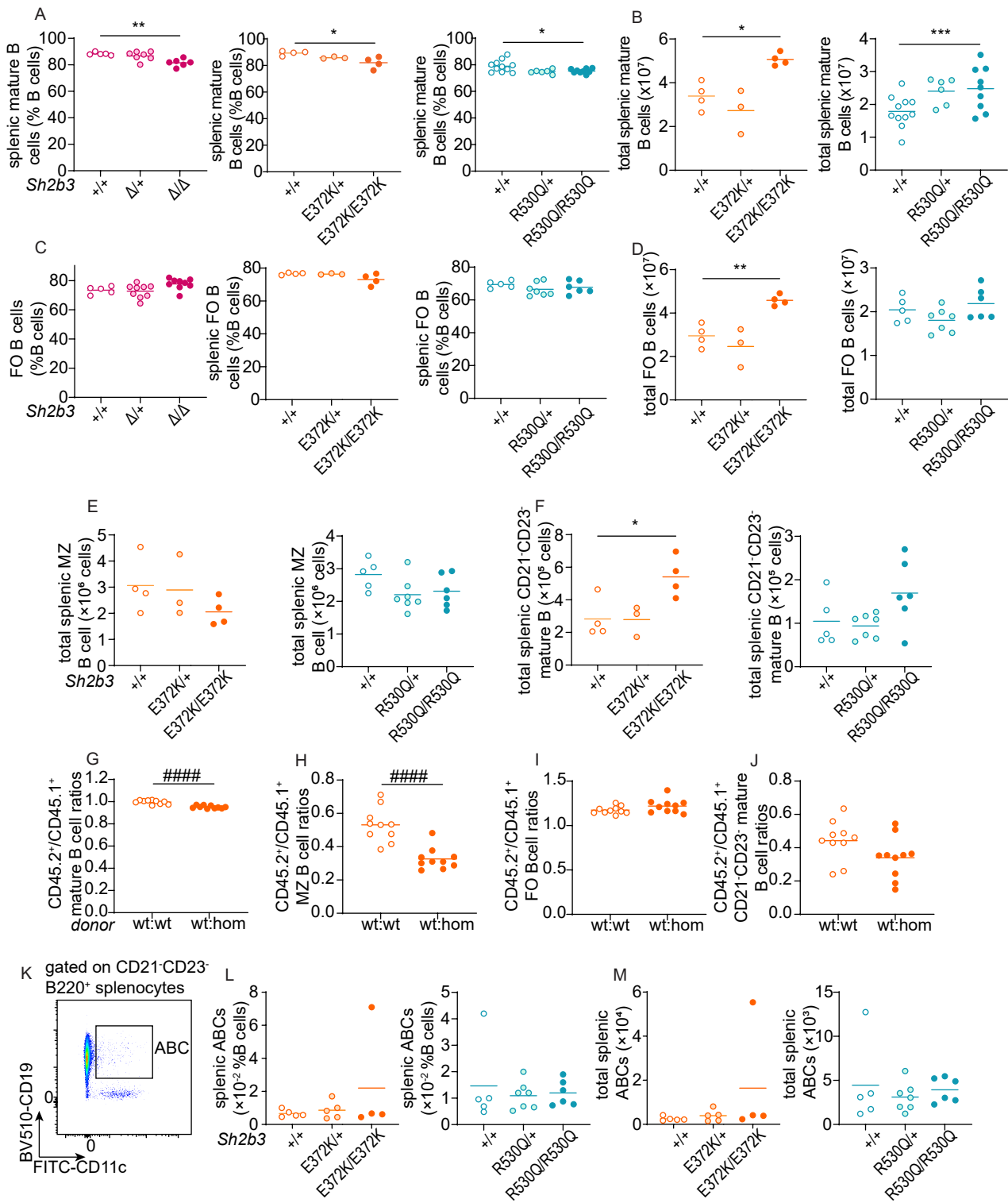

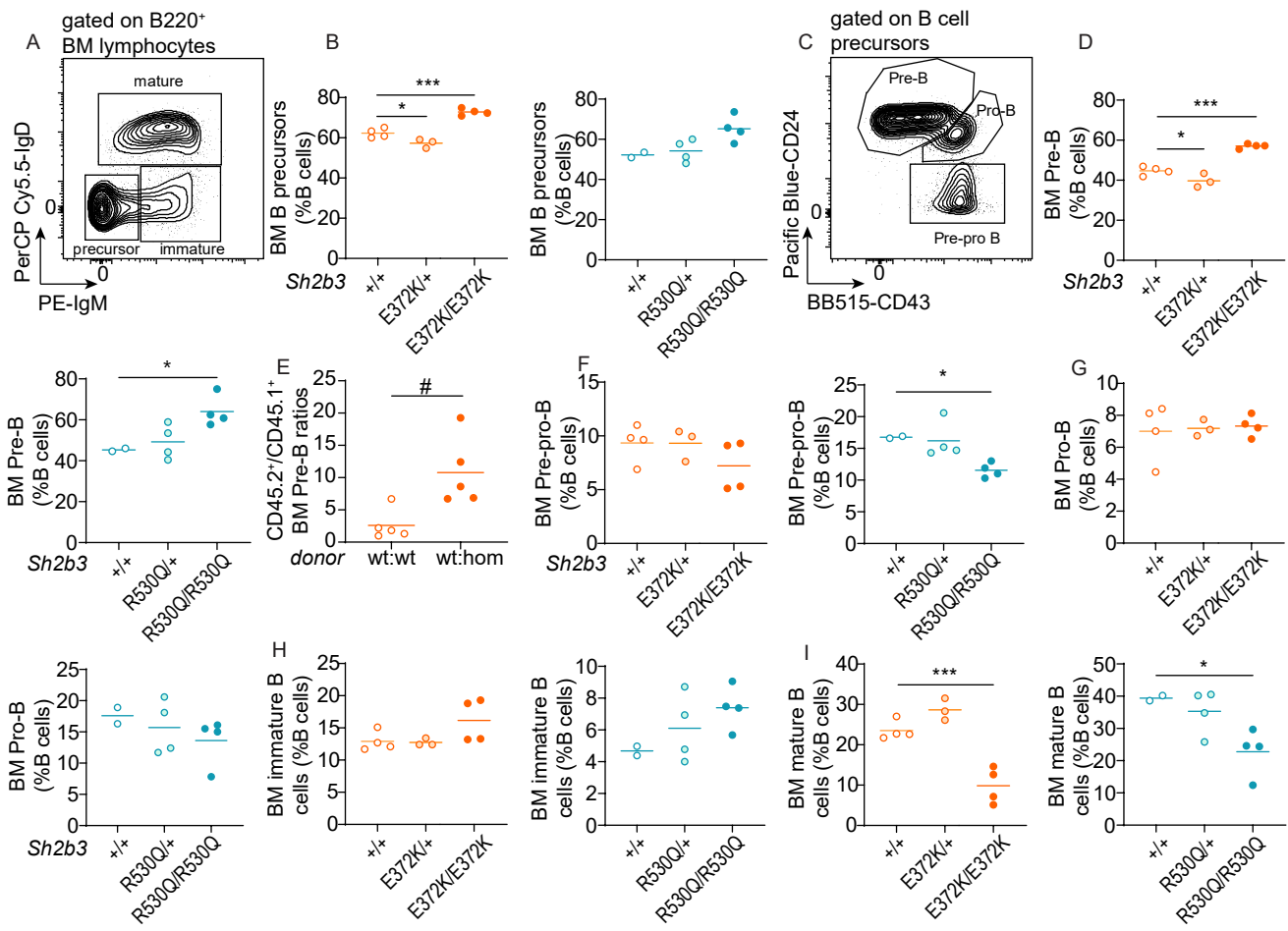

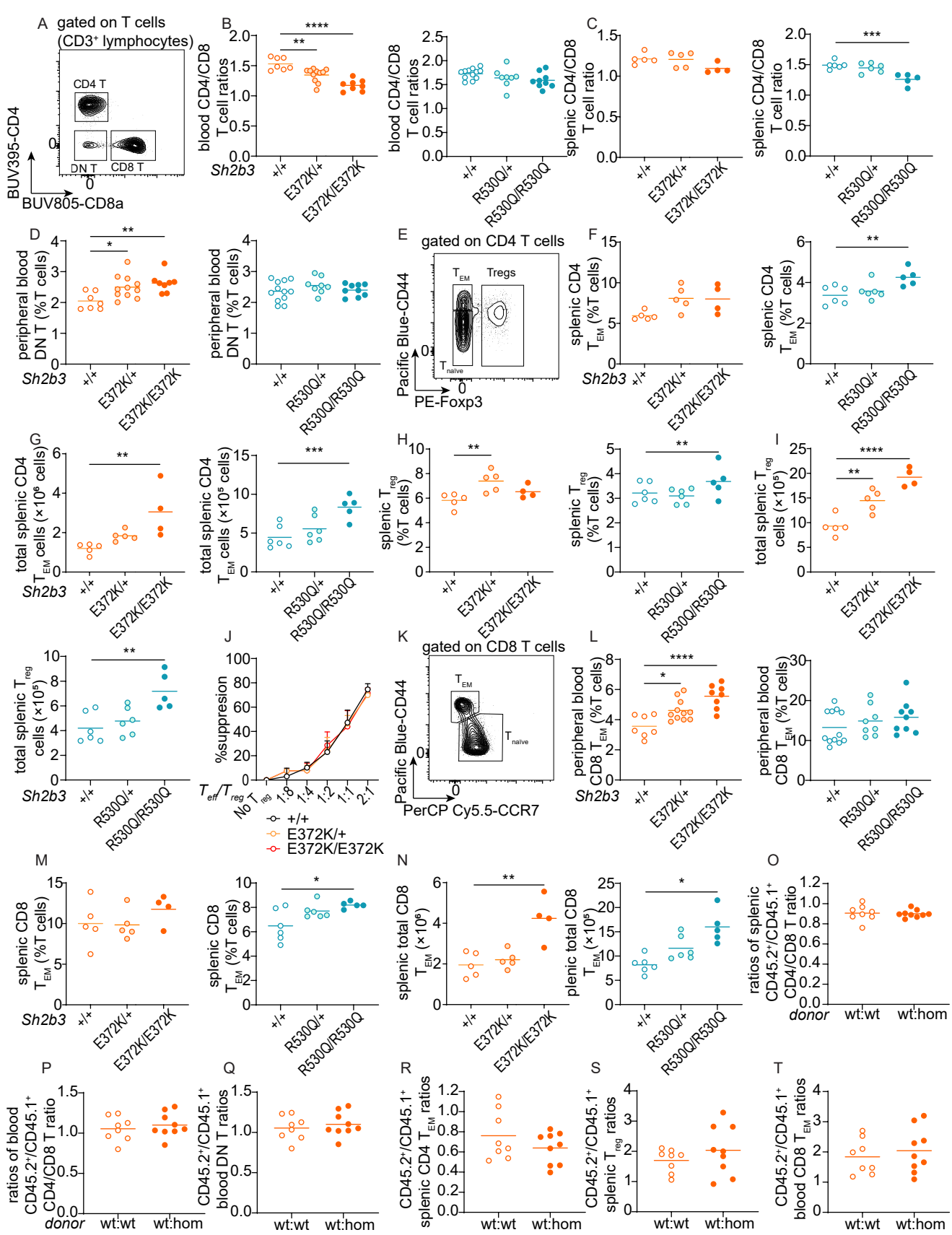

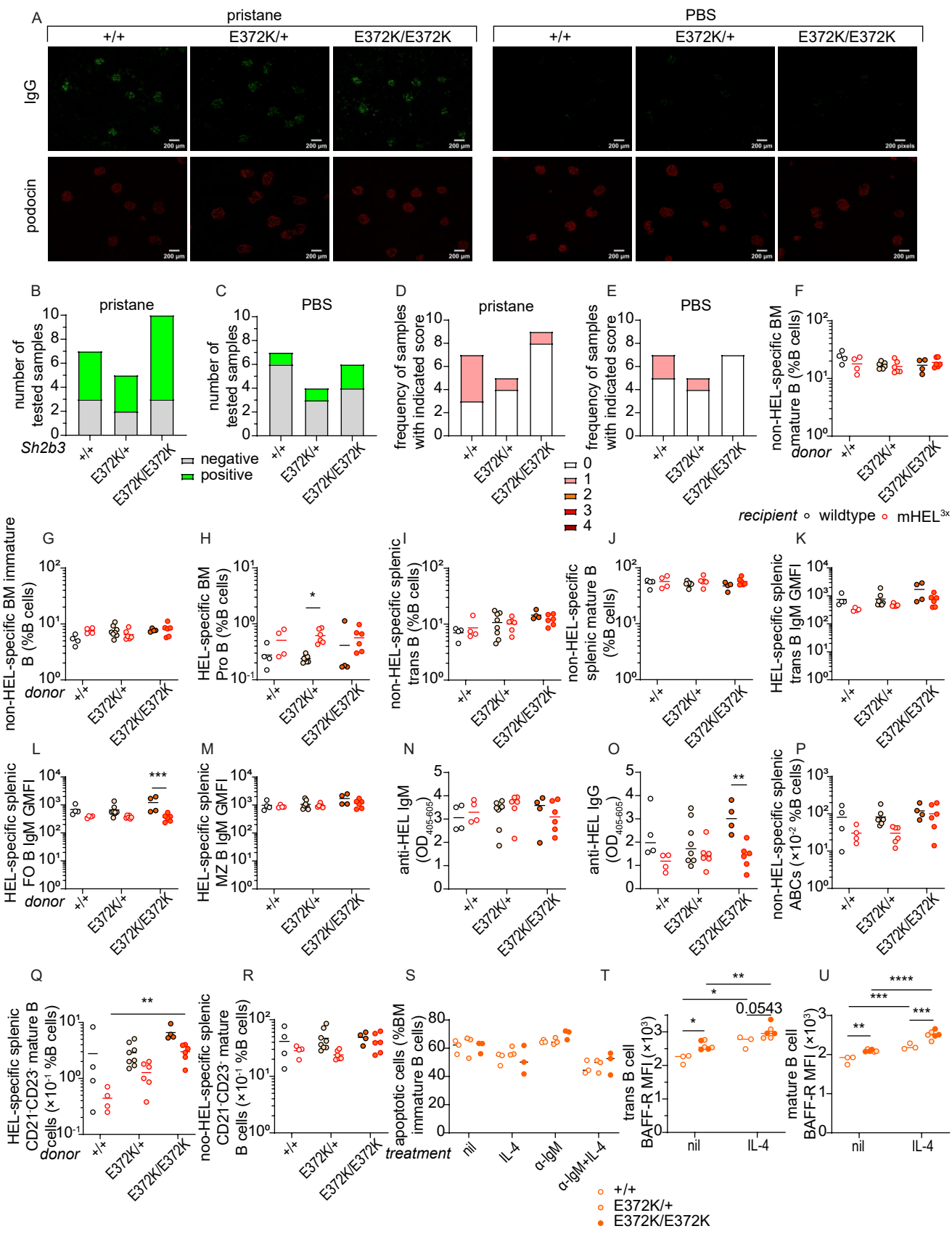

Supplement: Figure S1 [file EMS196089-supplement-Figure_S1.pdf]
