# Supplementary material for: Rare SH2B3 coding variants in lupus patients impair B cell tolerance and predispose to autoimmunity
Source: J Exp Med. Author manuscript; Available in PMC 2024 May 30. (PMC10901239; doi:10.1084/jem.20221080)
Supplement: Supplementary table 1 [file EMS196089-supplement-Supplementary_table_1.docx]

2

Table S1: Sex, ethnicity, onset, diagnosis and clinical manifestations of probands carrying *SH2B3* variants

| **Patient** | **Variant** | **Sex** | **Ethnicity** | **Age of SLE onset** | **Diagnosis** | **Clinical history** |
| --- | --- | --- | --- | --- | --- | --- |
| **A.II.1** | C133Y | F | Caucasian/  European | 15 | SLE with lupus nephritis, secondary immunodeficiency (hypogammaglobulinemia) and coagulopathy | **Postnatal**: dysmorphic features, failure to thrive, slow feeding habits, and physical delays.  **At the age of 15**: presented with fever, numerous spontaneous hematomas on the extremities and petechiae, swelling of the knees, polyarthralgias, frequent diarrheal stools accompanied by abdominal pain, inappetence and weight loss. Elevated erythrocyte sedimentation rate, pancytopenia, lymphopenia, coagulation disorder (prolonged prothrombin and activated partial thromboplastin time), proteinuria (0.64 g in 24-hr urine sample). Positive ANA in high titer (1:51200), positive anti-dsDNA, anti-histone and antiphospholipid antibodies (aPL), anti-cardiolipin (aCL) and lupus anticoagulant (LAC)) and low C3, C4 and CH50. Other laboratory findings included hypovitaminosis D3 and B12 and a high titer of antibodies to tissue trans- glutaminase. Renal biopsy was not performed. Enlarged liver with signs of diffuse lesion and splenomegaly was noticed on abdominal ultrasound, hypertrophic synovium of the elbows and knees with increased Doppler flow (synovitis) on diagnostic ultrasound of the locomotor system and retinal detachment of the left eye with maculopathy (dystrophy) of the right eye. She soon developed grand mal type epileptic seizures, without MRI signs typical for vasculitis of the central nervous system. The patient was treated with glucocorticoids (she received six intravenous pulses of glucocorticoids, followed by oral glucocorticoids) with hydroxychloroquine and azathioprine. She received antiepileptic drugs to establish satisfactory seizure control. Be- cause of hypogammaglobulinemia, she required replacement therapy with IVIG and because of severe CD4+ lymphopenia prophylaxis for opportunistic infections. Later she developed osteoporosis with multiple vertebral fractures (treated with bisphosphonates), arterial |
| Continued on next page | | | | | | |

3

| Table S1 – continued from previous page | | | | | | |
| --- | --- | --- | --- | --- | --- | --- |
| **Patient** | **Variant** | **Sex** | **Ethnicity** | **Age of SLE onset** | **Diagnosis** | **Clinical history** |
|  |  |  |  |  |  | hypertension and recurrent episodes of gastroenterocolitis accompanied by dehydration and metabolic acidosis. |
| **B.I.2** | E208Q | F | Caucasian  European | 33 | SLE, DVT | History of Hashimoto’s thyroiditis.  **At the age of 33:** diagnosis of SLE after longstanding history of mild photosensitive rashes and mild intermittent small joint arthralgias and sicca syndrome which was managed with hydroxychloroquine 200-400 mg daily alone. History of pulmonary emboli, deep vein thrombus and 2*×* miscarriage.  **At the age of 48:** diagnosed with antiphospholipid syndrome when border- line anticardiolipin antibody was detected. APLS was managed with regular warfarin.  **At the age of 62:** Patient self-ceased plaquenil due to concern re visual floaters. |
| **C.II.1** | E400K | M | Caucasian  English | 77 | SLE, DVT | **At the age of 73:** diagnosis of rheumatoid arthritis with small joint arthralgias, trialed on methotrexate.  **At the age of 77:** developed noninfectious, nonmalignant pleural effusion and noted ANA >2560 and elevated dsDNA antibody. Scl70 positive on ENA testing.  **At the age of 79:** diagnosed with bilateral DVT. |
| Continued on next page | | | | | | |

4

| Table S1 – continued from previous page | | | | | | |
| --- | --- | --- | --- | --- | --- | --- |
| **Patient** | **Variant** | **Sex** | **Ethnicity** | **Age of SLE onset** | **Diagnosis** | **Clinical history** |
| **D.II.2** | A536T | M | Chinese/  Asian | 9 | SLE, ITP, KD | **At the age of 6**: Diagnosed with ITP (presented with epistaxis and bruises),  treated with IVIG with good resolution of symptoms.  **At the age of 6**: presented with fever, rash, cervical lymphadenopathy, conjunctivitis and mucosal involvement and diagnosed with Kawasaki Disease. Treated with 2 courses of IVIG. Developed coronary artery involvement as part of Kawasaki Disease, experienced intermittent episodes of second- degree heart block (Mobitz type 1 with Wenckebach phenomenon).  **At the age of 9**: SLE diagnosed – treated with Methotrexate  **At the age of 10**: Presented with nephrotic syndrome and blood in urine and decreased kidney function – kidney biopsy showed class IV lupus nephritis – treated with steroids and mycophenolate with good response.  **At the age of 12**: Developed low mood, poor concentration and diagnosed with cerebral lupus – treated with IV methylprednisolone with resolution of the symptoms. |
| **E.II.1** | Q540X | M | Hispanic,  English/ European | 16 | SLE, ITP, DVT | **At the age of 16**: presented with severe SLE with ITP and secondary APS with DVT, Class IV glomerulonephritis, as well as skin and joint involvement. Positive anti-dsDNA, anti-Sm and anti-*β*2GPI antibodies, low serum complement. The patient was treated with cyclophosphamide, and maintained on 2000 mg/day mycophenolate, 400 mg/day hydroxychloroquine and warfarin. |
| Continued on next page | | | | | | |

5

| Table S1 – continued from previous page | | | | | | |
| --- | --- | --- | --- | --- | --- | --- |
| **Patient** | **Variant** | **Sex** | **Ethnicity** | **Age of SLE onset** | **Diagnosis** | **Clinical history** |
| **F.II.1** | R566Q | F | English | 29 | SLE with lupus nephritis | **At the age of 29**: presented with rash and polyarthritis, associated with renal involvement, elevated anti-dsDNA (1600 IU/ml), and positive anti-Ro60 antibodies. Also diagnosed with coexistent autoimmune thyroid disease with hypothyroidism, positive TPO antibodies. Renal biopsy revealed diffuse proliferative glomerulonephritis, for which she received 6 cycles of iv cyclophosphamide, maintained on a combination of mycophenolate mofetil, plaquenil and low dose prednisolone. She maintained clinical remission from later in the year onwards, but remained subclinically active with low C3, C4 and low-level proteinuria for many years; all of which have gradually improved. |
| **G.II.2** | R566Q | F | European | 12 | SLE | **At the age of 12**: presented with acute lupus featuring joint and muscle problems, required blood transfusions for haemolytic anaemia. Rental biopsy revealed glomerulonephritis. The patient was initially treated with mycophenolate, then with intravenous cyclophosphamide pulses for six months, which put her into remission and the treatment reverted to mycophenolate. The patient had high dose prednisolone until the age of 17. Despite remission, the patient had ongoing metacarpophalangeal joint and foot pain and swelling, as well as intermittent mouse ulcers. **At the age of 17**: normal joints, no evidence of skin rashes, serologically in remission (nor- mal complements, anti-dsDNA and ANAs), although experiences recurrent sinusitis and tonsillitis. The patient is still on mycophenolate. |
