# Supplementary material for: Rare SH2B3 coding variants in lupus patients impair B cell tolerance and predispose to autoimmunity
Source: J Exp Med. Author manuscript; Available in PMC 2024 May 30. (PMC10901239; doi:10.1084/jem.20221080)
Supplement: Supplementary table 2 [file EMS196089-supplement-Supplementary_table_2.docx]

Table S2: Rare variants in genes known to cause human SLE in the probands.

| Gene | A.II.1 | B.I.2 | C.II.1 | D.II.1 | E.II.1 | F.II.1 | G.II.2 |
| --- | --- | --- | --- | --- | --- | --- | --- |
| *C1QA* |  |  |  |  |  |  |  |
| *C1QB* |  |  |  |  |  |  |  |
| *C1QC* |  |  |  |  |  |  |  |
| *C1R* |  |  |  |  |  |  |  |
| *C1S* |  |  |  |  |  |  |  |
| *C2, CFB* |  |  |  |  |  |  |  |
| *C3* |  |  |  |  |  |  |  |
| *C4A* |  |  |  |  |  |  |  |
| *C4B* |  |  |  |  |  |  |  |
| *DNASE1* |  |  |  |  |  |  |  |
| *TREX1* |  |  |  |  |  |  |  |
| *PRKCD* |  |  |  |  |  |  |  |
| *DNASE1L3* |  |  |  |  |  |  |  |
| *ACP5* |  |  |  | p.G109R^1^  p.C238R^2^ |  |  |  |
| *TNFSF6* |  |  |  |  |  |  |  |
| *IFIH1* |  |  |  | p.A542E |  |  |  |
| *SOCS1* |  |  |  |  |  |  |  |
| *NCKAP1L* |  |  |  |  |  |  |  |
| *SAMHD1* |  |  |  |  |  |  |  |
| *ADAR1* | p.I939V |  |  |  |  |  |  |
| *RNASEH2B* |  |  |  |  |  |  |  |
| *TMEM173* |  |  |  |  |  |  |  |

^1^ ClinVar pathogenic variant

^2^ Novel variant
