# Supplementary material for: Rare SH2B3 coding variants in lupus patients impair B cell tolerance and predispose to autoimmunity
Source: J Exp Med. Author manuscript; Available in PMC 2024 May 30. (PMC10901239; doi:10.1084/jem.20221080)
Supplement: Supplementary table 3 [file EMS196089-supplement-Supplementary_table_3.docx]

Table S3: List of primers used for introducing patient-specific variants and other published variants into mammalian expression vectors of human *SH2B3*. Base change in each primer is in red.

| **Oligo Name** | **Sequence (5’** *→* **3’)** |
| --- | --- |
| h*SH2B3*sdmR43CFW | CGGGAGCTGGCC**T**GCCAGTACTGGC |
| h*SH2B3*sdmR43CRV | GCCAGTACTGGC**A**GGCCAGCTCCCG |
| h*SH2B3*sdmC133YFW | GCCCGGGCCCT**A**CTCCTTCCAGC |
| h*SH2B3*sdmC133YRV | GCTGGAAGGAG**T**AGGGCCCGGGC |
| h*SH2B3*sdmE208QFW | GCCTGGCCGAC**C**AGGCCTCCATG |
| h*SH2B3*sdmE208QRV | CATGGAGGCCT**G**GTCGGCCAGGC |
| h*SH2B3*sdmE400KFW | GACGCGGCGTGGG**A**AATACGTGCTCACT |
| h*SH2B3*sdmE400KRV | AGTGAGCACGTATT**T**CCCACGCCGCGTC |
| h*SH2B3*sdmA536TFW | CGCCCGAAGAACTG**A**CCAACAGCCTGCAG |
| h*SH2B3*sdmA536TRV | CTGCAGGCTGTTGG**T**CAGTTCTTCGGGCG |
| h*SH2B3*sdmQ540XFW | GGCCAACAGCCTG**T**AGCACCTGGAGCA |
| h*SH2B3*sdmQ540XRV | TGCTCCAGGTGCT**A**CAGGCTGTTGGCC |
| h*SH2B3*sdmR566QFW | CCGGAGCCACCTGC**A**GGCCATAGACAATC |
| h*SH2B3*sdmR566QRV | GATTGTCTATGGCC**T**GCAGGTGGCTCCGG |
