# Supplementary material for: Rare SH2B3 coding variants in lupus patients impair B cell tolerance and predispose to autoimmunity
Source: J Exp Med. Author manuscript; Available in PMC 2024 May 30. (PMC10901239; doi:10.1084/jem.20221080)
Supplement: Supplementary table 4 [file EMS196089-supplement-Supplementary_table_4.docx]

Table S4: Sequencing primers designed for validating ORF sequences in the mammalian expression vectors of human *SH2B3* via Sanger sequencing.

| **Oligo Name** | **Sequence (5’** *→* **3’)** |
| --- | --- |
| h*SH2B3*seqF1 | CTCCTCGCCCTCTTCC |
| h*SH2B3*seqF2 | GAAGTTCCTGCCCTGG |
| h*SH2B3*seqF3 | CATTTCCTGTCCTGCTACC |
| h*SH2B3*seqF4 | CAGAGGGTCTCCCAGG |
| h*SH2B3*seqR | CTGCAGCGACACCAG |
