# Supplementary material for: Rare SH2B3 coding variants in lupus patients impair B cell tolerance and predispose to autoimmunity
Source: J Exp Med. Author manuscript; Available in PMC 2024 May 30. (PMC10901239; doi:10.1084/jem.20221080)
Supplement: Supplementary table 5 [file EMS196089-supplement-Supplementary_table_5.docx]

Table S5: List of guide RNAs (gRNAs), single-stranded oligodeoxynucleotides (ssODNs) for CRISPR/Cas9 gene editing of mouse models, and oligos for validating the editing results by Sanger sequencing

| **Oligo Name** | **Sequence (5’** *→* **3’)** |
| --- | --- |
| *Sh*2*b*3*^R^*^530^*^Q^* gRNA | ACTGGTTGTCAATGGCC**C**GA |
| *Sh*2*b*3*^R^*^530^*^Q^* ssODN | AAATGGTTCCATTCACACGTCTGCCTCTCTGCACAGCTGTGAGAG AGGGGTGTACTGGTTGTCAATGGCC**T**GA**AGG**TGGCCCCGTGAAGA GGAGTCCATGTCATAGTCCGAGTCCCGGGCACTGCTCACAGACTC  GAGCTC |
| *Sh*2*b*3*^E^*^372^*^K^* gRNA | TGAGTACATACT**C**TCCTCTC |
| *Sh*2*b*3*^E^*^372^*^K^* ssODN | CCCCACCCTGGTCCACCAGTTCCTGCCCCTACCTTGGCTCTGCCC TGTAAGTTGAATGTGAGTACATACT**T**TCCTCTC**CGG**GACTCACTC TGCCGCACCAGGAACACGCCGTGGGCATCAGGGCCCTGGAGCTGG  ACCAGC |
| *Sh*2*b*3^∆^ gRNA | TGAGTACATACTCTCCTCTC |
| m*Sh*2*b*3*^R^*^530^*^Q^*seqFW | CTTGGTCTCTGGGTGTCCTT |
| m*Sh*2*b*3*^R^*^530^*^Q^*seqRV | GCACTGTCCACGCTCTGT |
| m*Sh*2*b*3*^E^*^372^*^K^*seqFW | CTTGGTCTCTGGGTGTCCTT |
| m*Sh*2*b*3*^E^*^372^*^K^*seqRV | GCACTGTCCACGCTCTGT |

PAM sequences are shown in bold text; single nucleotide variants are shown in green (wt) and red (mutant) with the affected amino acid codon sequence underlined.
