# Supplementary material for: Rare SH2B3 coding variants in lupus patients impair B cell tolerance and predispose to autoimmunity
Source: J Exp Med. Author manuscript; Available in PMC 2024 May 30. (PMC10901239; doi:10.1084/jem.20221080)
Supplement: Supplementary table 6 [file EMS196089-supplement-Supplementary_table_6.docx]

Table S6: Data collection and refinement statistics for the SH2 domains of murine SH2B3 protein with phosphopeptides bound

| E372K SH2B3 SH2 + JAK2 pY813 | |
| --- | --- |
| **Data Collection** | |
| Space group | C 2 |
| Cell dimensions  a, b, c (Å)  *α*, *β*, *γ* (°) | 75.82 37.61 50.49  90 108.76 90 |
| *R_meas_* (%) | 4.93 (76.49) |
| I /sigma(I) | 20.45 (2.49) |
| *CC*_1_*_/_*_2_ (%) | 99.90 (81.80) |
| Completeness (%) | 99.04 (97.32) |
| Redundancy | 6.8 (6.9) |
| **Refinement** | |
| Resolution (Å) | 34.53 - 1.64  (1.70 - 1.64) |
| No. reflections | 111266 (11000) |
| R-work | 17.9 (23.8) |
| R-free | 21.4 (26.9) |
| No. atoms | |
| protein | 914 |
| solvent | 76 |
| B factors | |
| protein | 32.45 |
| solvent | 43.76 |
| r.m.s. deviations | |
| RMS (bonds) | 0.006 |
| RMS (angles) | 0.85 |
| Ramachandran favored (%) | 95.41 |
| Ramachandran allowed (%) | 4.59 |
| Ramachandran outliers (%) | 0.00 |
| Rotamer outliers (%) | 2.08 |
