# Supplementary material for: Rare SH2B3 coding variants in lupus patients impair B cell tolerance and predispose to autoimmunity
Source: J Exp Med. Author manuscript; Available in PMC 2024 May 30. (PMC10901239; doi:10.1084/jem.20221080)
Supplement: Supplementary table 7 [file EMS196089-supplement-Supplementary_table_7.docx]

Table S7: Antibodies, fluorochrome-conjugated streptavidin, and viability dyes used in flow cytometry.

| **Reactivity** | **Antigen**  **/dye** | **Fluorochro-**  **me/ conjugate** | **Clone** | **Manufacturer** | **Cat. no** |
| --- | --- | --- | --- | --- | --- |
| mouse | CD16/32  (FcX) | purified | 93 | BioLegend | 101320 |
| anti-HEL  Ab | HEL (Ag) | purified | - | - | - |
| mouse | CD93 | biotin | AA4.1 | Invitrogen  (eBioscience) | 13-5892-85 |
| mouse | CD138 | biotin | 281-2 | BD Pharmingen | 553713 |
| mouse | CXCR5 | biotin | 2G8 | BD Pharmingen | 551960 |
| mouse | B220 | biotin | RA3-6B2 | BioLegend | 103204 |
| mouse | B220 | BUV395 | RA3-6B2 | BD Horizon | 563793 |
| mouse | CD4 | BUV395 | GK1.5 | BD Horizon | 563790 |
| mouse | CD19 | BUV395 | 1D3 | BD Horizon | 563557 |
| mouse | CD23 | BV421 | B3B4 | BioLegend | 101621 |
| mouse | I-A/I-E  (MHCII) | BV421 | M5/114.15  .2 | BioLegend | 107631 |
| mouse | PD-1 | BV421 | 29F-1A12 | BioLegend | 135218 |
| phosphatidy-  lserine | Annexin V | Pacific Blue | - | BioLegend | 640918 |
| mouse | CD24 | Pacific Blue | M1/69 | BioLegend | 101820 |
| mouse | CD44 | Pacific Blue | IM7 | BioLegend | 103020 |
| amine | Live/dead  fixable aqua | - | - | Invitrogen | L34965 |
| mouse | CD93 | BV480 | AA4.1 | BD  Optibuild | 746239 |
| mouse | CD19 | BV510 | eBio1D3 | Invitrogen  (eBioscience) | 56-0193-82 |
| mouse | CD95 | BV510 | Jo2 | BD Horizon | 563646 |
| biotin | Streptavidin | BV510 | - | BioLegend | 405234 |
| phosphatidy-  lserine | Annexin V | FITC | - | BD  Pharmingen | 556419 |
| Continued on next page | | | | | |

Table S7 – continued from previous page

| **Reactivity** | **Antigen**  **/dye** | **Fluorochro-**  **me/ conjugate** | **Clone** | **Manufacturer** | **Cat. no** |
| --- | --- | --- | --- | --- | --- |
| mouse | BAFF-R | FITC | eBio71-  122-E16 | Invitrogen  (eBioscience) | 11-5943-81 |
| mouse | CD11b | FITC | M1/70 | BD  Pharmingen | 7222717 |
| mouse | CD11c | FITC | N418 | BioLegend | 117306 |
| mouse | CD62L | FITC | H1.2F3 | BD  Pharmingen | 553236 |
| mouse | Foxp3 | FITC | FJK-16s | Invitrogen  (eBioscience) | 2007700 |
| mouse | IgM | FITC | II/41 | BD  Pharmingen | 553437 |
| mouse | CD34 | BB515 | MEC14.7 | BioLegend | 119302 |
| mouse | BST2  (CD317) | PE | 927 | BioLegend | 127009 |
| mouse | CD3 | PE | 17A2 | BioLegend | 100206 |
| mouse | CD4 | PE | RM4-5 | eBioscience | 12-0042-83 |
| mouse | CD25 | PE | PC61 | BioLegend | 102008 |
| HEL | HyHEL9 | PE | HyHEL9 | prepared by  P.F. Cañete | - |
| mouse | Foxp3 | PE | FJK-16s | Invitrogen  (eBioscience) | 4323635 |
| mouse | IgD | PE | 11-26c-2a | BioLegend | 405706 |
| mouse | Ig*λ* | PE | RML-42 | BioLegend | 407308 |
| mouse | PD-1 | PE | J43 | Invitrogen  (eBioscience) | 4293188 |
| mouse | CD21/35 | BV605 | 7G6 | BD Horizon | 583176 |
| mouse | CD43 | BV605 | S7 | BD Horizon | 563205 |
| mouse | CD45.1 | BV605 | A20 | BioLegend | 110737 |
| mouse | CD86 | BV605 | GL1 | BD Horizon | 563055 |
| mouse | CD138 | BV605 | 281-2 | BioLegend | 142515 |
| biotin | Streptavidin | BV605 | - | BioLegend | 405229 |
| mouse | CD23 | BV711 | B3B4 | BD Horizon | 563987 |
| DNA | 7-AAD | 7-AAD | - | Invitrogen | A1310 |
| mouse | CCR7 | PerCP-  Cy5.5 | 4B12 | BioLegend | 120116 |
| Continued on next page | | | | | |

Table S7 – continued from previous page

| **Reactivity** | **Antigen**  **/dye** | **Fluorochro-me/ conjugate** | **Clone** | **Manufacturer** | **Cat. no** |
| --- | --- | --- | --- | --- | --- |
| mouse | CD4 | PerCP-  Cy5.5 | RM4-5 | BioLegend | 100540 |
| mouse | IgD | PerCP-  Cy5.5 | 11-26c.2a | BD  Pharmingen | 564273 |
| mouse | CD4 | PE-Cy7 | RM4-5 | BD  Pharmingen | 552775 |
| mouse | CD98 | PE-Cy7 | RI.388 | BioLegend | 128214 |
| mouse | IgD | PE-Cy7 | 11-26c.2a | eBioscience | 25-5993-82 |
| mouse | IgM | PE-Cy7 | II/41 | Invitrogen  (eBioscience) | 25-5790-81 |
| mouse | KLRG1 | PE-Cy7 | 2F1 | eBioscience | 25-5893-82 |
| biotin | Streptavidin | PE-Cy7 | - | BioLegend | 405206 |
| mouse | CD45.1 | APC | 104 | BioLegend | 109814 |
| mouse | SiglecH | APC | 551 | BioLegend | 129611 |
| mouse | Bcl6 | Alexa Fluor  647 | K112-91 | BD  Pharmingen | 561525 |
| mouse | CD4 | Alexa Fluor  647 | RM4-5 | BioLegend | 100530 |
| mouse | CD11c | Alexa Fluor  647 | N418 | BioLegend | 117312 |
| mouse | CD25 | Alexa Fluor  647 | PC61 | BioLegend | 102020 |
| HEL | HyHEL9 | Alexa Fluor  647 | HyHEL9 | Prepared by  P. F. Cañete | - |
| amine | Fixable  viability dye eFluor780 | eFluor780 | - | Invitrogen  (eBioscience) | 65-0865-14 |
| mouse | CD3 | Alexa Fluor  700 | 17A2 | BioLegend | 100216 |
| mouse | CD4 | Alexa Fluor  700 | RM4-5 | BD  Pharmingen | 557956 |
| mouse | CD19 | Alexa Fluor  700 | eBio1D3 | Invitrogen  (eBioscience) | 56-0193-82 |
| mouse | Ig*κ* | Alexa Fluor  700 | RMK-45 | BioLegend | 409508 |
| Continued on next page | | | | | |

Table S7 – continued from previous page

| **Reactivity** | **Antigen**  **/dye** | **Fluorochro-**  **me/ conjugate** | **Clone** | **Manufacturer** | **Cat. no** |
| --- | --- | --- | --- | --- | --- |
| mouse | CD45.2 | BUV737 | 104 | BD Horizon | 564880 |
| mouse | CD8a | BUV805 | 53-6.7 | BD Horizon | 564920 |
| human | FcX | purified | 3G8 (CD16), FUN-2 (CD32), 10.1 (CD64) | BioLegend | 422302 |
| human | IgM | eFluor450 | SADA4 | Invitrogen  (eBioscience) | 48-9998 |
| human | IgD | BV510 | IA6-2 | BioLegend | 348220 |
| human | CD24 | BV605 | MLS | BioLegend | 311124 |
| human | CD19 | BV605 | HIB19 | BioLegend | 302238 |
| human | CD38 | PerCP-  Cy5.5 | HIT2 | BD  Pharmingen | 551400 |
| human | IgA | PE | IS11-8E10 | Miltenyi Biotech | 130-093-  128 |
| human | CD10 | PE-CF594 | HI10a | BD Horizon | 562396 |
| human | IgG | PE-Cy7 | G18-145 | BD  Pharmingen | 561298 |
| human | CD21 | APC | B-ly4 | BD  Pharmingen | 561767 |
| human | CD27 | APC-  eFluor780 | O323 | Invitrogen  (eBioscience) | 47-0279 |
