# Supplementary material for: Rare SH2B3 coding variants in lupus patients impair B cell tolerance and predispose to autoimmunity
Source: J Exp Med. Author manuscript; Available in PMC 2024 May 30. (PMC10901239; doi:10.1084/jem.20221080)
Supplement: Supplementary table 8 [file EMS196089-supplement-Supplementary_table_8.docx]

Table S8: Primary and secondary antibodies used for ELISA and immunofluorescence.

| **Host** | **Reactivity** | **Antigen** | **Conjugate** | **Clone** | **Manufac-**  **turer** | **Cat. no** |
| --- | --- | --- | --- | --- | --- | --- |
| **ELISA** | | | | | | |
| goat | mouse | IgM | human  ads-AP | polyclonal | Southern  Biotech | 1020-04 |
| goat | mouse | IgG | human  ads-AP | polyclonal | Southern  Biotech | 1030-04 |
| **Immunofluorescence** | | | | | | |
| **Host** | **Reactivity** | **Antigen** | **Conjugate** | **Clone** | **Manufac-**  **turer** | **Cat. no** |
| goat | mouse | podocin | purified | C-18 | Santa  Cruz Biotech- nology | sc-22296 |
| donkey | mouse | IgG (H+L) | Alexa  Fluor 488 | polyclonal | Invitrogen | A-21202 |
| donkey | goat | IgG (H+L) | Alexa  Fluor 594 | polyclonal | Invitrogen | A-11058 |
