# Supplementary material for: Rare SH2B3 coding variants in lupus patients impair B cell tolerance and predispose to autoimmunity
Source: J Exp Med. Author manuscript; Available in PMC 2024 May 30. (PMC10901239; doi:10.1084/jem.20221080)
Supplement: Supplementary Figure Caption [file EMS196089-supplement-Supplementary_Figure_Caption.docx]

**Legends for Supplementary Figures**

**Figure S1. Human and murine SH2B3 protein functionality and gross phenotypes of *Sh2b3^Δ^*, *Sh2b3^E372K^* and *Sh2b3^R530Q^* mice and transitional B cell phenotypes in *Sh2b3^E372K^* and *Sh2b3^R530Q^* mice, and chimeras of *Sh2b3^E372K^* mice.**(**A**) Relative GAS activity of unstimulated HEK293 cells overexpressing wildtype or *SH2B3*variants identified in SLE and HC cohorts. Sample numbers for each condition is listed as follow: empty (*n* = 21), WT (*n* = 21), R43C (*n* = 9), C133Y (*n* = 12), E208Q (*n* = 21), E400K (*n* = 15), A536T (*n* = 18), Q540X (*n* = 18), R566Q (*n* = 21). Means are shown as bars and all conditions were compared to cells transfected with WT SH2B3. Linear mixed-effect model (lmer) with estimated marginal means (emmeans) using experiment as the blocking factor was used for the statistical analyses. ***: p < 0.001, ****: p < 0.0001.(**B**) Relative GAS activity of HEK293T cells overexpressing wildtype or *SH2B3* variants listed in gnomAD database at MAF>0.0005in presence of IFN-γ (50 ng/mL) stimulation.Results are pooled from 4 independent experiments and shown as the percentage of GAS activity compared to the EV control. Each dot is a biological replicate and the mean of three technical replicates.Resultsare pooled from five independent experiments. Sample numbers for each condition is listed as follow: empty (n = 30), WT (n = 11), W262R (n = 9), T165S (n = 9), F182L (n = 12), S186I (n = 12), P242S (n = 12), R80C (n = 12), A36E (n = 9), E78K (n = 9).Linear mixed-effect model (lmer) with estimated marginal means (emmeans) using experiment as the blocking factor was used for the statistical analyses. ****p < 0.0001.(**C**) Representative direct binding curves for the WT and E372K SH2B3 SH2 domains to a JAK2 pY813 peptide. Results are representative of X experiments. (**D**) Melting temperatures for WT (unfilled) and E372K (filled)SH2B3 SH2 domains in their apo form and after the addition of the phosphomimetic phenyl phosphate (PP), JAK2 pY813, JAK3 pY785 and EPOR pY454 peptides. *n*= 3-7. Means are shown as bars.Two-way ANOVA with with Šídák's adjustmentwas used for statistical analyses. *p < 0.05, **p < 0.01, ***p < 0.001, ****p < 0.0001.Results are representative of X experiments. (**E**) Representative flow cytometric plot showing the gating strategy of B cells (CD19^+^CD3^-^) and T cells (CD19^-^CD3^+^). (**F and G**) Dotplots showing frequencies ofB(F) and T cells (G) as percentages of total splenic lymphocytes in *Sh2b3^E372K^* (upper panel) and *Sh2b3^R530Q^* (lower panel) mice. (**Hand I**) Total numbers of splenic transitional B cells(H) and T1 B cells (I)in *Sh2b3^E372K^*(left) and *Sh2b3^R530Q^*(right) mice. (**J**) CD45.2^+^/CD45.1^+^ T1 B cell ratios among splenic B cells in 50:50 BM chimeras of CD45.1-*Sh2b3^+/+^* and CD45.2-*Sh2b3^+/+^*/*Sh2b3^E372K/E372K^* mice. (**K**) Frequencies of T2 B cells as percentages of splenic B cells in *Sh2b3^Δ^* (left)*,Sh2b3^E372K^*(middle) and *Sh2b3^R530Q^*(right) mice. (**L**) Total numbers of splenic T2 B cells in *Sh2b3^E372K^* (left) and *Sh2b3^R530Q^* (right) mice. (**M**) CD45.2^+^/CD45.1^+^ T2 B cell ratios among splenic B cells in 50:50 BM chimeras of CD45.1-*Sh2b3^+/+^* and CD45.2-*Sh2b3^+/+^*/*Sh2b3^E372K/E372K^* mice. (**N**) Frequencies of T3 B cells as percentages of splenic B cells in *Sh2b3^Δ^* (left),*Sh2b3^E372K^* (middle) and *Sh2b3^R530Q^* (right) mice.O)Total numbers of splenic T3 B cells in *Sh2b3^E372K^* and *Sh2b3^R530Q^* mice. Frequencies of (**P**) CD45.2^+^/CD45.1^+^ T3 B cell ratios among splenic B cells in 50:50 BM chimeras of CD45.1-*Sh2b3^+/+^* and CD45.2-*Sh2b3^+/+^*/*Sh2b3^E372K/E372K^* mice.Results in F-Pare representative of 2-3 independent experiments.Each dot represents one mouse.Sample numbers for each group in F-I, K, L, N and O are listed as follows, *Sh2b3^Δ^* panel: +/+ (*n* = 5), Δ/+ (*n* = 7), Δ/Δ (*n* = 6);*Sh2b3^E372K^* panel: +/+ (*n* = 5 in F and G, *n* = 4 in others), E372K/+ (*n* = 5 in F and G, n = 3 in others), E372K/E372K (*n* = 4); *Sh2b3^R530Q^* panels: +/+ (n = 6 in F and G, n = 5 in others), R530Q/+ (n = 6 in F-H, n = 7 in others), R530Q/R530Q (n = 5-9). N = 10 in J and P.Means are indicated as bars. Student-t tests were used for the statistical analysis in D, J, M and P. One-way ANOVA was used for the statistical analyses in F-I, K, L, N and O. Significance levels in lmer ANOVAs are indicated with asterisks, while those in multiple student-t tests are indicated with hashes. */#: p < 0.05, **/##: p < 0.01, ***/###: p < 0.001, ****/####: p < 0.0001.

**Figure S2.Mature B cell phenotypes in*Sh2b3^Δ^*,*Sh2b3^E372K^* and *Sh2b3^R530Q^* mice, and chimeras of *Sh2b3^E372K^* mice.** (**A**) Frequencies of mature B cells as percentages of splenic B cells. (**B**) Total numbers of splenic mature B cells. (**C**) Frequencies of FO B cells as percentages of splenic B cells. (**D**) Total splenic FO B cells. (**E and F**) Total splenic MZ B cells (E) and CD21^-^CD23^-^ mature B cells(F). Each dot represents one mouse.Sample numbers for each group in A-F are listed as follow, *Sh2b3^Δ^* panels: +/+ (*n* = 5), Δ/+ (*n* = 7), Δ/Δ (*n*= 6); *Sh2b3^E372K^* panels: +/+ (*n* = 4), E372K/+ (*n* = 3), E372K/E372K (*n* = 4); *Sh2b3^R530Q^* panels: +/+ (*n* = 5-11), R530Q/+ (*n* = 6-7), R530Q/R530Q (*n* = 6-9).Means are shown as bars. One-way ANOVA was used for statistical analysis. (**G-J**) CD45.2^+^/CD45.1^+^ ratios of frequencies of splenicmature B cells(G), MZ B cells(H), FO B cells (I) and CD21/35^-^CD23^-^ mature B cells(J) in 50:50 BM chimeras of CD45.1-*Sh2b3^+/+^* and CD45.2-*Sh2b3^+/+^*(*n* = 10) or *Sh2b3^E372K/E372K^*(*n* = 10)mice. Statistical analysis performed using student t-test. Results are representative of two independent experiments. (**K**)Representative flow cytometric plot of splenic atypical memory B cells (ABCs; B220^+^CD21/35^-^CD23^-^CD11c^+^CD19^+^). (**L and M**) Frequencies (L) and total numbers (M) of splenic ABCs. Results are representative of 2-3 independent experiments.Means are shown as bars. One-way ANOVA was used for statistical analysis of immunophenotyping data (A-F, L and M) while student t-test was used for analyzing data from BM chimera experiments (G-J). Significance levels of one-way ANOVAs are indicated with asterisks while those of student-t tests are indicated with hashes. Significance level criteria are indicated as follow: */#: p < 0.05, **/##: p < 0.01, ***/###: p < 0.001, ****/####: p < 0.0001.

**Figure S3.BM B cell phenotypes in *Sh2b3^E372K^* and *Sh2b3^R530Q^* mice.A-I**. BM B cell phenotypes.(**A**) Representative flow cytometric plot showing the gating of B cell precursors (IgM^-^IgD^-^), immature (IgM^+^IgD^-^) and mature (IgD^+^) B cells. Cells were pregated on B220^+^ lymphocytes. (**B**) Frequencies of B cell precursors as percentages of BM B cells. (**C**) Representative flow cytometric plot showing the gating of pre-pro (CD24^-^CD43^+^), pro- (CD24^hi^CD43^+^) and pre-B cells (CD24^+^CD43^-/lo^).Frequencies of pre-B cells as percentages of BM B cells in (**D**) *Sh2b3^E372K^*(left) and *Sh2b3^R530Q^* (right) mice and (**E**) 50:50 BM chimeras of CD45.1-*Sh2b3^+/+^* and CD45.2-*Sh2b3^+/+^*/*Sh2b3^E372K/E372K^* mice(*n* = 5). Frequencies of (**F**) pre-pro and (**G**) pro-, (**H**) immature and (**I**) mature cells as percentages of BM B cells.Lines in all dot plots show means and results are representative of two independent experiments.Sample numbers for each group in B, D, F-M are listed as follow, *Sh2b3^E372K^* panels: +/+ (*n* = 4), E372K/+ (*n* = 3), E372K/E372K (n = 4); *Sh2b3^R530Q^* panels: +/+ (*n* = 2), R530Q/+ (*n* = 4), R530Q/R530Q (*n* = 4). One-way ANOVA was used for statistical analysis of immunophenotyping data (B, D, F-M),student t-test was used for analyzing data from BM chimera experiments (E). Significance levels of one-way ANOVAs are indicated with asterisks while those of student t-testsare indicated with hashes. Significance level criteria are indicated as follow: */#: p < 0.05, **/##: p < 0.01, ***/###: p < 0.001, ****/####: p < 0.0001.

**Figure S 4.Peripheral blood and splenic T cell phenotypes in *Sh2b3^E372K^* and *Sh2b3^R530Q^* mice.**(**A**) Representative flow cytometric plot showing the gating of double-negative (DN; CD4^-^CD8^-^), CD4^+^ (CD4^+^CD8^-^) and CD8^+^ (CD4^-^CD8^+^) T cells. Dot plots showing the ratios of (**B**) blood and (**C**) splenic CD4/CD8 T cells in *Sh2b3^E372K^* mice and splenic CD4/CD8 T cells in *Sh2b3^R530Q^* mice. (**D**) Frequencies of DN T cells in the peripheral blood of *Sh2b3^E372K^* mice. (**E**) Representative flow cytometric plot showing the gating of CD4 naïve T (T_naïve_: CD44^lo/-^Foxp3^-^), effector memory T (T_EM_: CD44^hi^Foxp3^-^) and regulatory T (T_reg_: Foxp3^+^) cells. (**F**) Frequencies and (**G**) total numbers of CD4 T_EM_ cells in the spleens of *Sh2b3^E372K^* and *Sh2b3^R530Q^* mice. (**H**) Frequencies and (**I**) total numbers ofof T_reg_ cells in the spleen of *Sh2b3^E372K^* (left) and *Sh2b3^R530Q^* (right) mice. (**J**)Percentage suppression of effector T cells (T_eff_) by T_reg_cellsin culture at various T_eff_/T_reg_ ratios by using T_reg_cellssorted from the spleens of *Sh2b3^+/+^*(*n* = 3), *Sh2b3^E372K/+^*(*n* = 3)and *Sh2b3^E372K/E372K^*(*n* = 4)mice. (**K**) Representative flow cytometric plot showing the gating of CD8 T_naïve_ (CD44^lo/-^CCR7^lo^) and T_EM_ (CD44^hi^CCR7^-^) cells. Frequencies of (**L**) peripheral blood and (**M**) splenic CD8 T_EM_ cells in *Sh2b3^E372K^* and*Sh2b3^R530Q^* mice. (**N**) Total numbers of splenic CD8 T_EM_ in *Sh2b3^E372K^* and *Sh2b3^R530Q^* mice. Ratios of CD45.2^+^/CD45.1^-^ (**O**) splenic and (**P**) peripheral blood CD4/CD8 T ratios, (**Q**) peripheral blood DN T cells, (**R**) splenic T_EM_ cells, (**S**) T_reg_cells and (**T**) peripheral blood CD8 T_EM_ cellsin 50:50 BM chimeras of CD45.1-*Sh2b3^+/+^* and CD45.2-*Sh2b3^+/+^*/*Sh2b3^E372K/E372K^* mice(*n* = 8).Results in B-D, F-J, L-N and Sare representative oftwo independent experiments. Results inO-R and T are from a single experiment.Sample numbers for each group in these panels are listed as follow, *Sh2b3^E372K^* panels: +/+ (spleen: *n* = 5, blood: *n* = 7), E372K/+ (spleen: *n* = 5, blood: *n* = 11), E372K/E372K (spleen: *n* = 4, blood: *n* = 9); *Sh2b3^R530Q^* panels: +/+ (spleen: *n* = 6, blood: *n*=12), R530Q/+ (spleen: *n* = 6, blood: *n*=8), R530Q/R530Q (spleen: *n* = 5, blood: *n*=9).One-way ANOVA was used for statistical analysis of immunophenotyping data (B-D, F-I and L-N) while student-t test was used for analyzing data from BM chimera experiments (O-T). Significance levels of one-way ANOVAs are indicated with asterisks while those of student-t tests are indicated with hashes. Significance level criteria are indicated as follow: */#: p < 0.05, **/##: p < 0.01, ***/###: p < 0.001, ****/####: p < 0.0001.

**Figure S5. Spontaneous autoimmunity and B cell tolerance in *Sh2b3^E372K^* mice. A-C**. IgG immune-complex deposits in mice treated with PBS or pristane. (**A**) Representative immunofluorescence images showing IgG (green) and podocin (red) staining in the kidney sections of pristane/PBS-treated *Sh2b3^E372K^* mice 20 weeks following treatment with pristane and PBS. Numbers of kidney sections that are positive and negative for IgG ICs in the glomeruli of *Sh2b3^E372K^*mice treated with (**B**) pristane(+/+: *n* = 7, E372K/+: *n* = 5, E372K/E372K: *n* = 10) and (**C**) PBS(+/+: *n* = 7, E372K/+: *n* = 4, E372K/E372K: *n* = 6).**D-E.** Numbers of kidney sections with indicated glomerular score 20 weeks following treatment with (**D**) pristane (+/+: *n* = 7, E372K/+: *n* = 5, E372K/E372K: *n* = 9)and (**E**) PBS(+/+: *n* = 7, E372K/+: *n* = 5, E372K/E372K: *n* = 7).**F-R**. Cellular and serological phenotypes of SW_HEL_-mHEL^3×^ chimeric mice.Mouse numbers for each group are listed as follows: +/+ wildtype (*n* = 4), +/+ mHEL^3×^ (*n* = 4), E372K/+ wildtype (*n* = 8), E273K/+ mHEL^3×^ (*n* = 6), E372K/E372K wildtype (*n* = 4), E372K/E372K mHEL3× (*n* = 6). Frequencies of non-HEL-specific BM (**F**) mature and (**G**) immature B cells, (**H**) HEL-specific Pro-B cells, non-HEL-specific splenic (**I**) transitional and (**J**) mature B cells, relative surface IgM expression on HEL-specific splenic (**K**) transitional, (**L**) FO and(**M**) MZ B cellsin WT (black circles) or mHEL^3×^ (red circles) recipients receiving BM from SW_HEL_-*Sh2b3^+/+^* (unfilled), SW_HEL_-*Sh2b3^E372K/+^* (yellow filling) or SW_HEL_-*Sh2b3^E372K/E372K^* (orange filling) donors. Levels of anti-HEL (**N**) IgM and (**O**) IgG in wildtype and mHEL^3×^ mice receiving BM from SW_HEL_-*Sh2b3^+/+^*, -*Sh2b3^E372K/+^* and -*Sh2b3^E372K/E372K^* donors measured as OD_405-605_ by ELISA. Frequencies of (**P**) non-HEL-specific ABCs,(**Q**) HEL-specificand (**R**) non-HEL-specific CD21^-^CD23^-^ mature B cellsas percentages of splenic lymphocytes. (**S**) Percentages of apoptotic BM immature B cells in untreated, 20 ng/mL IL-4 only, 5 μg/mL anti-IgM (α-IgM) only, and 5 μg/mL α-IgM + 20 ng/mL IL-4 conditions(*n* = 3).BAFF-R surface expression on (**T**) transitional and (**U**) mature B cellsmeasured by flow cytometry as median fluorescence intensity (MFI)(*n* = 3).Data in A-Cis pooled from two independent experiments. Results in D-Uare representative of 2-3 independent experiments. Fisher’s exact test was used for the statistical analyses in B-E. Means in D-Uare shown as bars, two-way ANOVA was used for statistical analysis in G-U. Significance levels for two-way ANOVAs are indicated with asterisks. *: p < 0.05, **: p < 0.01, ***: p < 0.001, ****: p < 0.0001.
